# How about running on Mars? Influence of sensorimotor coherence on running and spatial perception in simulated reduced gravity

**DOI:** 10.3389/fphys.2023.1201253

**Published:** 2023-07-31

**Authors:** Marie Keime, Loïc Chomienne, Cédric Goulon, Patrick Sainton, Thomas Lapole, Rémy Casanova, Martin Bossard, Caroline Nicol, Cécile Martha, Benoit Bolmont, Arnaud Hays, Fabrice Vercruyssen, Pascale Chavet, Lionel Bringoux

**Affiliations:** ^1^ Aix Marseille University, CNRS, ISM, Marseille, France; ^2^ École Centrale Marseille, Marseille, France; ^3^ KTH, Stockholm, Sweden; ^4^ Université Jean Monnet Saint-Etienne, Lyon 1, Université Savoie Mont-Blanc, Laboratoire Interuniversitaire de Biologie de la Motricité, Saint-Etienne, France; ^5^ University Gustave Eiffel, COSYS-PICS-L, Marne-la-Vallée, France; ^6^ University of Lorraine, 2LPN-CEMA Group, Metz, France; ^7^ University of Toulon, IAPS, Toulon, France

**Keywords:** motor adaptation, gravity, space analog, locomotion, sensory integration, spatial perception, unweighting

## Abstract

Motor control, including locomotion, strongly depends on the gravitational field. Recent developments such as lower-body positive pressure treadmills (LBPPT) have enabled studies on Earth about the effects of reduced body weight (BW) on walking and running, up to 60% BW. The present experiment was set up to further investigate adaptations to a more naturalistic simulated hypogravity, mimicking a Martian environment with additional visual information during running sessions on LBPPT. Twenty-nine participants performed three sessions of four successive five-min runs at preferred speed, alternating Earth- or simulated Mars-like gravity (100% vs. 38% BW). They were displayed visual scenes using a virtual reality headset to assess the effects of coherent visual flow while running. Running performance was characterized by normal ground reaction force and pelvic accelerations. The perceived upright and vection (visually-induced self-motion sensation)in dynamic visual environments were also investigated at the end of the different sessions. We found that BW reduction induced biomechanical adaptations independently of the visual context. Active peak force and stance time decreased, while flight time increased. Strong inter-individual differences in braking and push-off times appeared at 38% BW, which were not systematically observed in our previous studies at 80% and 60% BW. Additionally, the importance given to dynamic visual cues in the perceived upright diminished at 38% BW, suggesting an increased reliance on the egocentric body axis as a reference for verticality when the visual context is fully coherent with the previous locomotor activity. Also, while vection was found to decrease in case of a coherent visuomotor coupling at 100% BW (i.e., post-exposure influence), it remained unaffected by the visual context at 38% BW. Overall, our findings suggested that locomotor and perceptual adaptations were not similarly impacted, depending on the -simulated- gravity condition and visual context.

## 1 Introduction

“That’s one small step for man, one giant leap for mankind,” stated Neil Armstrong when he set foot on the Moon in 1969. Fifty years later, the goal of space exploration remains similar: land, walk and ultimately, settle, on another planet. With this purpose in mind, future missions will have to take into account the adaptive abilities of the astronauts to keep them in optimal conditions during the journey and settlement on a novel environment, Mars being the current target.

So far, human adaptation to space missions has mostly been evaluated in microgravity during parabolic flights and on the International Space Station (ISS). Some studies (see White et al., 2020 for a review) focused on sensorimotor skills, i.e., motor production based on sensory state estimates and feedback, mainly examined through reaching movements (Bringoux et al., 2012; Bringoux et al., 2020; Gaveau et al., 2016; Macaluso et al., 2017), grasping tasks (Crevecoeur et al., 2010; Giard et al., 2015) or more complex tasks such as “reach to lift” (Patron et al., 2002) or bouncing series (Ritzmann et al., 2016). All the aforementioned studies have highlighted specific sensorimotor adaptations to microgravity. Among these previous works, some quantified adaptive effects of exposure to different visual flow patterns during and after walking (e.g., Mulavara et al., 2005; Nomura et al., 2005; Richards et al., 2007), but not running. It was reported that heading direction and locomotor trajectory were notably affected. Surprisingly however, while running or skipping may constitute a mechanically fitting pattern of locomotion when the level of gravity is reduced (Cavagna et al., 1972; Pavei et al., 2015), there is, to our knowledge, no corresponding study which tested running adaptation. In fact, the effects of a reduced level of gravity on locomotion have been scarcely investigated, be it on trained astronauts or on novice participants, partly because of the lack of adapted experimental analogs. Among recent technologies, the LBPP treadmill (LBPPT) provides body weight (BW) support (i.e., partial unweighting) as the air pressure increases within the associated flexible chamber, hence creating a lifting force from the pelvic level (Whalen and Hargens, 1992). Such analog appears thus promising as it gives the possibility, on Earth, to study locomotion under simulated hypogravity conditions (Fazzari et al., 2023).

A primary consequence of unweighting on locomotion, specifically during running, is the previously reported adoption of a longer flight duration combined with a reduced duration of the contact phase leading to the slowing of stride frequency (Grabowski and Kram, 2008; Sainton et al., 2015; Farina et al., 2017). Such a pattern recalls skipping adopted by Neil Armstrong on the Moon. Our previous studies precisely characterized the running pattern at 80% and 60% BW on a LBPPT, bringing forth that biomechanical adaptations to hypogravity are specific to both the unweighting level and the running phase (Sainton et al., 2015). However the presence of after-effects on the reloading phase reveals that the temporal adaptations of the running pattern are not optimal (Sainton et al., 2015). A potential explanation for these discrepancies could be the lack of visual information often reported as a factor favouring a default pattern (Liebermann and Goodman, 1991; Müller et al., 2014).

The aim of the present study was to test the influence of visual information, notably through the manipulation of visual cues in terms of sensorimotor coupling, on running adaptation in a realistic Mars-like environment. Besides, we also questioned how spatial perception evolves in response to these various sensory contexts. Until now, only 80% and 60% BW levels have been studied using a LBPPT, while studies about spatial perception were mostly performed on the ISS and on parabolic flights. It is yet unknown whether adaptations are comparable under Martian gravity (around 38% BW) which might be experienced in the near future.

In the following experiment, we studied locomotor adaptations and related spatial perception following manipulation of the multisensory context during running. By multisensory context, we consider two BW levels mimicking Earth and Mars gravity (100% or 38% BW respectively) and different related visual information. We hypothesized that visual context would influence locomotor adaptations to simulated hypogravity, such that a coherent visual scene, which traduces the natural head oscillations and forward translations when running in hypogravity, might induce more noticeable adaptations of the running pattern. Our second hypothesis was that spatial perception in simulated hypogravity would also be influenced by visual context. The latter was manipulated during running along the vertical axis to subsequently study its impact on upright orientation, and along the anteroposterior axis, to evaluate the effects on visually-induced forward self-motion perception (i.e., vection). We expected that simulated hypogravity would decrease the reliance on visual cues for upright perception and suppress post-exposure effect on vection in case of a coherent visuo-locomotor activity.

## 2 Methods

### 2.1 Participants

Twenty-seven healthy male recreational runners (mean age 19.6 ± 1.8 years) volunteered for this study. Individuals with counter-indications to running, with medical/surgical antecedents (motor and sensory issues) or any illness/injury not compatible with the study (affecting lower limbs, spine, and sensory inputs) were automatically excluded. For technical reasons, a participant was unable to perform the spatial tests following his running sessions. This study received approval by the ethics committee of the ethical national instance CERSTAPS (IRB00012476-2021-31-03-96). In accordance with the Declaration of Helsinki, all participants provided written informed consent to take part in the experiment.

### 2.2 Materials

**FIGURE 1:**
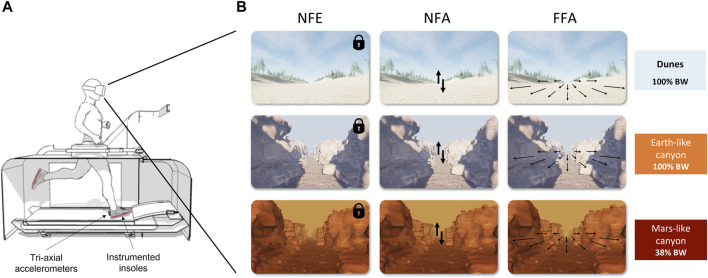
**(A)** Sketch of the experimental setup including the LBPPT used for the experiment. The differential air pressure technology allowed for controllable unweighting of the runner on the treadmill. Participants were equipped with accelerometers and wore shoes with specific insoles to collect information about the running pattern. They wore a VR headset displaying different visual scenes along the experiment. **(B)** illustration of the visual scenes displayed when running 1) in PRE and POST sessions (upper panel); 2) in PER session at 100% Body Weight (middle panel); 3) in PER session at 38% Body Weight (lower panel). The different visual conditions manipulating the reference frames/optic flow presented to the participants (NFE, NFA, and FFA) were displayed from the left to right respectively.

The experiments were conducted at the Institute of Movement Science in Marseille (France). Each volunteer ran on a LBPPT (VIA400X AlterG®, Fremont, CA, United States; Figure 1A) wearing neoprene shorts zipped to the flexible chamber of the treadmill, at the height of the greater trochanter. Initial inside chamber air (100% BW) was pressurized to reach 38% BW, simulating hypogravity (0.38 g). 100% BW thus corresponded to normogravity (1 g).

#### 2.2.1 Biomechanical measurements

Two tri-axial accelerometers (Pico Cometa systems®, Milan, Italy, ±8 G, F = 2 kHz) were positioned on the right foot, one on the posterior aspect of the heel, one medially (navicular), and a third one was placed at the sacrum level. Data was recorded through the software EMGandMotionTools®. Instrumented insoles (Loadsol, Novel®, Munich, Germany; F = 100 Hz) were inserted in each running shoe (Run active, Kalenji®) to record the normal ground reaction force (GRF) on posterior, antero-medial and antero-lateral surface of the foot. Data was acquired using the Loadsol application software. Head position was acquired through the sensors of the HP Reverb G2 virtual reality (VR) headset (F = 90 Hz, 2160 × 2160 pixels per eye). The VR headset was also used to display the different visual scenes during the running sessions and to perform spatial tests.

#### 2.2.2 Visual conditions

##### 2.2.2.1 Visual scenes and flows

To display the different visual scenes, the participants wore a HP Reverb G2 VR headset controlled by a laptop (Windows Mixed Reality, Steam VR, using Unreal Engine to develop the scenes). The onsets of both accelerometer acquisition and visual scenes were synchronized with the help of an external controller (Leo Bodnar Electronics, BU0836A 12-Bit Joystick Controller). Throughout a test session, participants were successively presented with three different visual scenes: one that was clearly taking place on Earth, with a visual background different from the others to avoid habituation, and two picturing a canyon, respectively with Earth-like and with Mars-like features (e.g., sky color, pathway texture, see Figure 1B). Prior to each test session, the visual scene was calibrated to the participant’s height. Participants ran and went through the whole session with the headset on.

Different visual conditions (i.e., reference frames/visual flow) were presented to each participant, in a pseudo-randomized order over three sessions.

• No Flow Ego (NFE): static viewpoint anchored to the participant (egocentric fixation). The image did not change regardless of participant’s head movements.
• No Flow Allo (NFA): static viewpoint anchored to the external environment (allocentric fixation). The participant could look around the scene as if he was standing still in a room.
• Full Flow Allo (FFA): allocentric anchor (same as above) with a dynamic viewpoint and a continuous retinal flow at the same speed as that of the treadmill corresponding to each participant’s preferred speed to give a realistic running impression. In this condition, the focus of expansion characterizing the motion direction of the visual scene mimicking a forward displacement was always kept centred at eye level with respect to the external space (ahead of the participant’s initial natural orientation on the treadmill).

##### 2.2.2.2 Spatial tests

**FIGURE 2:**
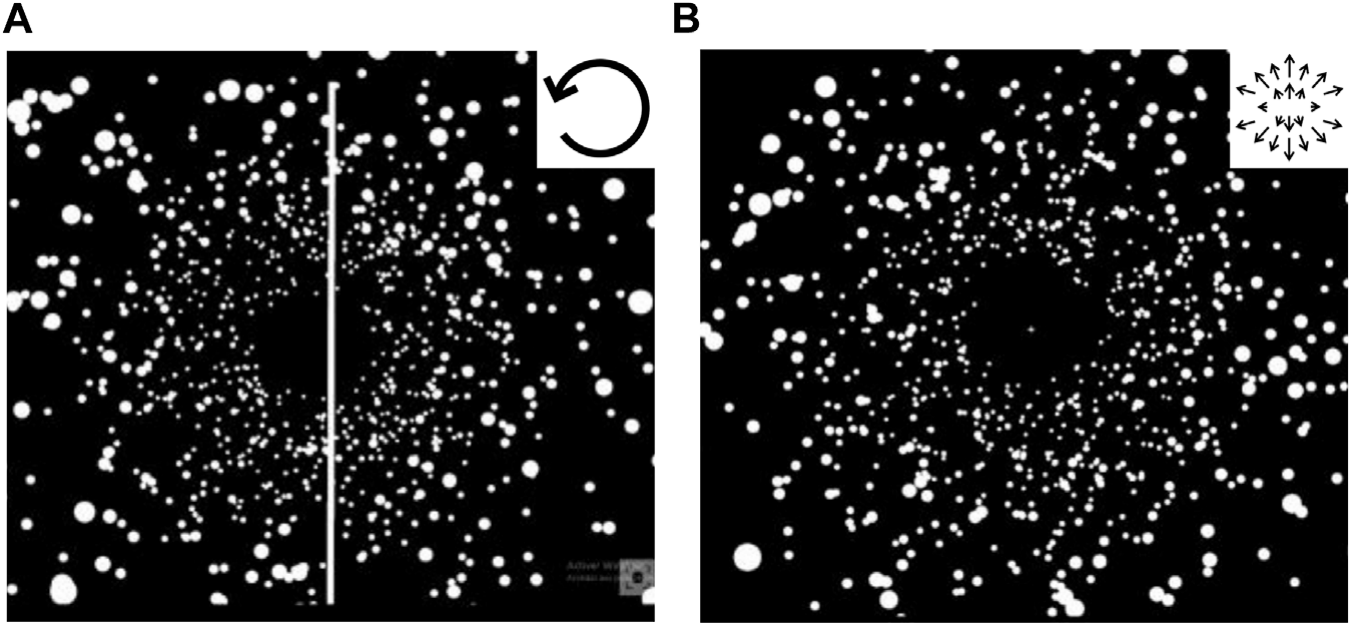
**(A)** Snap of the visual scene displayed between each run session to assess the perceived upright orientation (Rod and Disc Test). Participants were asked to continuously align the rod to their subjective upright (using a joystick) while facing a rotating cloud of dots in the frontal plane during 30 s. **(B)** Snap of the visual scene displayed between each run session to assess vection. Participants were asked to estimate the “quantity” of visually-induced forward self-motion (using a joystick) while facing an expanding cloud of dots at eye level along the sagittal axis during 30 s. The motion direction of the clouds of dots displayed for each test was presented in the top right corner of each panel.

Two tests were used to evaluate the interplay between visual inputs and locomotor adaptations through the consequences on spatial perception. Visuals of the tests were developed in-house by using Unreal Engine 5.0 and displayed using the VR headset, with responses given by the participants using the right joystick (F = 90 Hz). The participants stood still throughout the tests without laying their resting hand on the treadmill. The Rod-and-disk test (RDT) was first presented to evaluate the dynamic visual influence on the perceived upright orientation (Dichgans et al., 1972). The use of VR technology for assessing upright perception has been found valuable in static (RFT: Bringoux et al., 2009) and dynamic (RDT: Zaleski-King et al., 2020) virtual environments. Using the right joystick of the VR set, the participants were asked to continuously adjust a rod to upright in the presence of a rotating visual flow (white points on a black background moving at 30°/s, Figure 2A). The joystick could be pushed to tilt the rod accordingly to the left or right. At the beginning of the test, the rod was upright (i.e., aligned to gravity). The test was designed to give the participants the illusion that the rod was rotating, while only the background was moving. The more influenced the participants were by the rotating background, the more they moved the joystick and re-adjusted the position of the rod. In the second spatial test, the participants had to continuously evaluate their visually-induced forward self-motion perception (i.e., vection) while viewing an antero-posterior visual flow (expanding cloud of points on a black background, 10 m.s^−1^, Figure 2B). Vection was defined to the participants as the sensation of moving forward when facing visual stimuli moving backward while there is no actual physical motion occurring. They were asked to move the joystick along the antero-posterior axis to evaluate how intensely they felt self-motion: the more intense the feeling, the more they pushed the joystick forward.

### 2.3 Procedure

#### 2.3.1 Familiarization

One week before the main session, each volunteer participated in a familiarization session to get a first experience of the LBPPT and determine their preferred running speed. They first filled in the VIMSSQ questionnaire (Keshavarz et al., 2019) to assess their tolerance to VR and long-time screen exposure, before trying on the VR headset. From this questionnaire, we did not spot any participants too highly susceptible to VR sickness to participate.

#### 2.3.2 Tests

**FIGURE 3:**
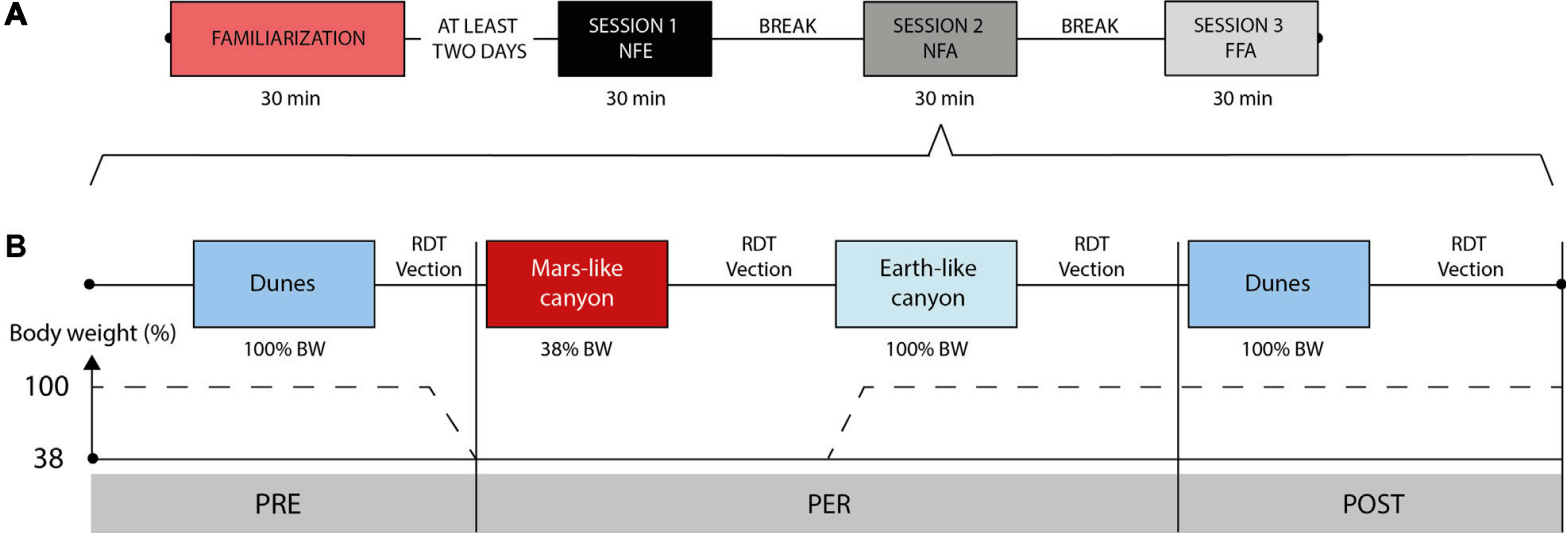
**(A)** Sketch of the protocol with the 3 running sessions preceded by the familiarization session, and example order of visual conditions (here: NFE–NFA–FFA). **(B)** Example of a detailed session presenting a specific visual condition (e.g., NFA). Half the participants began the PER phase with the 38% BW run, as pictured, the other half started with the 100% BW run.

Each selected participant went through three test sessions (each presenting a different visual condition), performed over the same day (4 h). Each session was divided in PRE, PER, and POST phases (Figure 3). The PRE and POST runs were conducted at 100% BW while displaying in the VR headset a common Earth-based scene (dunes) in FFA condition. The PER phase was composed of two runs, at 38% and 100% BW, presented in a counterbalanced manner. They were successively performed under the same visual condition, either NFE, NFA or FFA. The order of the sessions including one of the three visual conditions in the PER phase was pseudo-randomized.

Each session thus presented four consecutive runs of 5-min duration each, performed at the participant’s preferred running speed established during familiarization with the VR headset (on average, 10.5 ± 0.6 km/h). The same running speed per participant was maintained across each run for comparison purpose. Although it is known that the preferred running speed on a treadmill could be impacted by the available visual flow (Prokop et al., 1997), it was found comfortable and natural across the whole experiment.

Each run pre-started with the treadmill being gradually accelerated up to the preferred running speed during approximately 30 s. The visual scene was displayed in the VR headset as soon as the participant started running, thus allowing gradual immersive experience proper to each visual condition before entering each 5-min run at constant speed. Each run was followed by RDT and vection tests. Both tests were successively presented as soon as the participant had stopped running and was stable and ready to enter this perceptual evaluation phase.

### 2.4 Data analysis

All data analyses were carried out on Matlab R2021b (MathWorks, Inc.). Acceleration signals were low-pass filtered using a 4th order Butterworth zero-phase filter with a 10 Hz cut-off frequency. The vertical acceleration recorded at the pelvic level was double integrated and band-pass filtered to identify the end of the braking phase, corresponding to the minimum vertical position of the pelvis. Active peak force (APF), contact and toe-off points were determined using the normal component of the GRF, with a force detection threshold set at 50 N. Flight, stride, stance, step frequency, braking and push-off durations were then computed as well as the amplitude of the pelvic vertical displacement during stance (i.e., during braking (Δ*H*_B_) and during push-off (Δ*H*_P_)). Maximum rod deviation during RDT (MaxDev) along the 30 s duration of the test was chosen as the variable of interest for the first spatial test. This variable represents the maximum amount of disturbance on the perceived upright orientation encountered by the participants due to the rotating visual field. Quantity of vection (QVec) was the variable selected for the second spatial test and was obtained by integrating vection intensity recorded over the 30 s duration of the test, after applying a low-pass filter (1 Hz). This variable thus reflects a single perceptual effect combining both magnitude and duration of visually-induced self-motion perception, allowing to test the complex experience of vection in a more holistic way (Kooijman et al., 2023).

### 2.5 Statistics

Statistical analyses were conducted using JASP software (version 0.16.3) with a level of significance set to *p* < 0.05. Outliers with inconsistent and extremely high results on vection and RDT data were removed from the analysis. For each variable, two repeated measures Analysis of Variances (ANOVAs) were performed. The first specifically compared the different phases (PRE; PER38; PER100; POST) with a repeated single visual condition (FFA) to stress the unweighting effect itself. Also, a 2-BW level [PER100; PER38] × 3 visual conditions [NFE; NFA; FFA] repeated measures ANOVA was performed to focus on the interaction between the visual coherence relative to the sensorimotor activity and the BW condition. A Greenhouse-Geissner sphericity correction was applied to the ANOVA results when necessary. Significant effects were further examined using post-hoc tests with Bonferroni correction. The results were expressed as means and standard deviations.

## 3 Results

Data analysis focused first on the effects of the simulated gravity condition (i.e., BW influence across experimental phases) independently of the visual conditions, before also considering the latter during PER exposure to bring forward possible interactions between gravitational and visual contexts.

### 3.1 Effects of body weight reduction on the running pattern

The ANOVA performed over the 4 phases (PRE, PER100, PER38, POST) revealed a significant influence of the BW condition on the running pattern. The results of ANOVAs conducted on each running parameters are synthesized in Table 1.

**TABLE 1:** Variations of biomechanical parameters between PER (38% BW) and PRE (100% BW) runs.

Variable	Values (PRE - PER38)	% Variation/PRE runs	Statistical significance
Stance Time	0.292 s – 0.250 s	−14%	F_(3,26)_ = 191.1; *p* < 0.001; qp^2^ = 0.880
Step Frequency	2.501 step/s to 2.041 step/s	−18%	F_(3,26)_ = 265.018; *p* < 0.001; qp^2^ = 0.911
Braking time	0.164 s – 0.158 s	−4%	F_(3,26)_ = 0.621; *p* = 0.573; qp^2^ = 0.023 (ns)
Push-off time	0.154 s – 0.139 s	−11%	F_(3,26)_ = 0.709; *p* = 0.529; rip^2^ = 0.027 (ns)
Pelvic vertical displacement during braking (ΔHB)	0.018 m – 0.012 m	−33%	F_(3,26)_ = 9.865; *p* < 0.001; qp^2^ = 0.275
Pelvic vertical displacement during push-off (ΔHP)	0.021 m – 0.011 m	−48%	F_(3,26)_ = 22.744; *p* < 0.001; qp^2^ = 0.532
Active Peak Force	1686 N – 998 N	−41%	F_(3,26)_ = 550.787; *p* < 0.001; ripe = 0.957
Flight time	0.088 s – 0.213 s	142%	F_(3,26)_ = 440.42; *p* < 0.001; qp^2^ = 0.944

**FIGURE 4:**
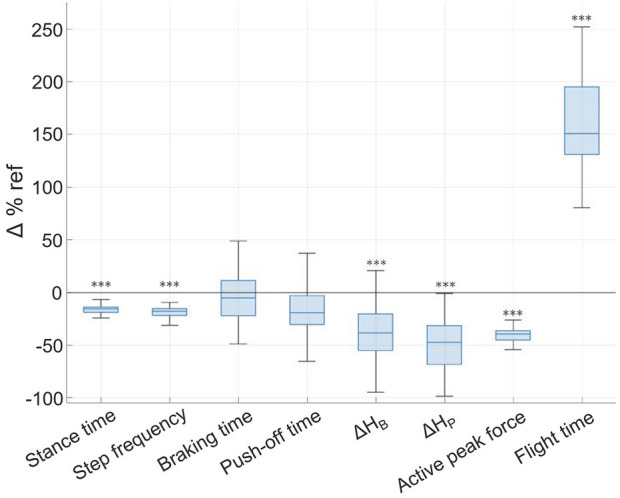
Relative unweighting-induced variations (Δ%ref) of the temporal characteristics of the left and right steps. For each variable the individual changes are represented by the median, interquartile range, and extreme values. ***p < 0.001 when statistically different from the initial condition values during PRE run.

Unweighting condition (PER38), specifically compared to each other phase, led to longer flight time, shorter stance time and lower step frequency (*p* < 0.001). The relative difference between the parameters at 100% BW (during PRE runs) and at 38% BW (during PER38 runs) led to a decreased stance time that went along with the decreased step frequency, while flight time increased by 147%, consequently enough to stand out of the other values (Figure 4). Interestingly, braking and push-off durations did not significantly differ between phases despite the decreased stance duration.

Δ*H*_B_, Δ*H*_P_, and APF were also reduced by 30–40% at 38% BW compared to 100% BW. In other words, there was less vertical pelvic excursion during braking and push-off and, upon contact, the ground normal reaction force was lessened with unweighting.

### 3.2 Effects of body weight reduction on upright perception

**FIGURE 5:**
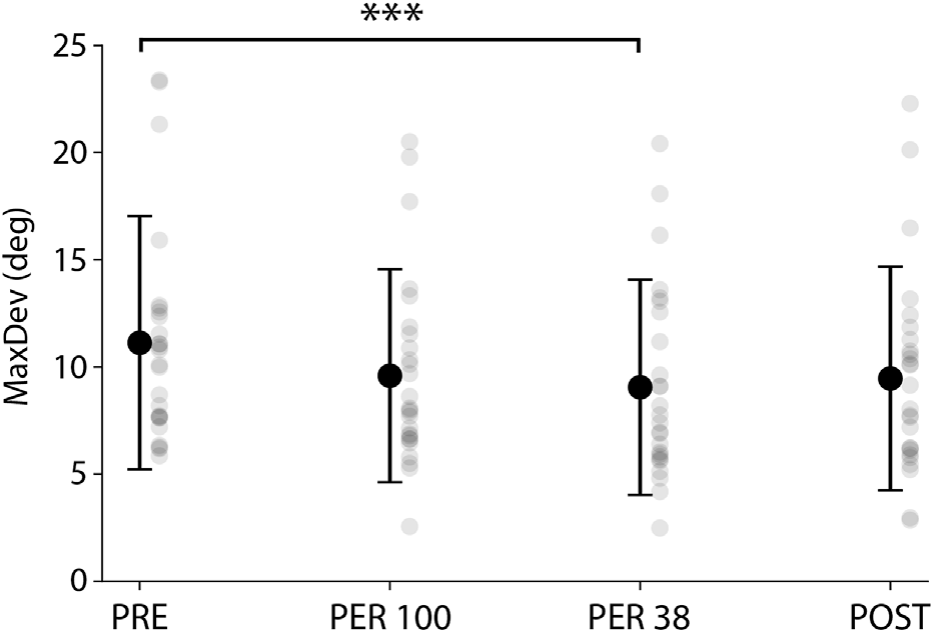
MaxDev (Mean maximum deviation of the rod assessed to upright perception) over the 4 phases. ***p < 0.001.

Body weight reduction not only affected the running pattern as it also tampered with the participants’ spatial perception, as shown by the results of the RDT revealing how upright perception is significantly influenced by a dynamic visual environment following each phase (F_(3, 25)_ = 5.66; *p* < 0.01; ηp^2^ = 0.19). MaxDev decreased significantly in PER38 compared to PRE [−25% (11.3°–8.4°), *p* < 0.001; Figure 5]. Thus, it appears that the maximum deviation recorded during the RDT following a 38% BW run was significantly smaller than when the same test was performed after the very first run at 100% BW.

### 3.3 Effects of body weight reduction on vection

Vection (QVec) was barely but not significantly impacted by the BW condition (F_(3,25)_ = 2.78; *p* = 0.053; ηp^2^ = 0.10, after Greenhouse-Geissner correction). While some participants were immune to any visually-induced motion stimuli throughout the whole experiment, some others presented high responses, particularly when Qvec was evaluated following a run at 38% BW.

### 3.4 Interaction between gravitational and visual contexts

The previous subsections presented the results pertaining to the experimental phases to stress the effect of body weight reduction only, yet our study also introduced varying visual contexts during the two PER runs, at 38% and 100% BW. Our goal was here to focus on a possible interaction between the visual context (NFE; NFA; FFA) and the simulated gravity (i.e., loading conditions: PER100; PER38).

We did not observe any significant effect of the visual condition nor any interaction between the visual and loading conditions on the biomechanical parameters (*p* > 0.05 for the eight parameters characterizing the running pattern).

**FIGURE 6:**
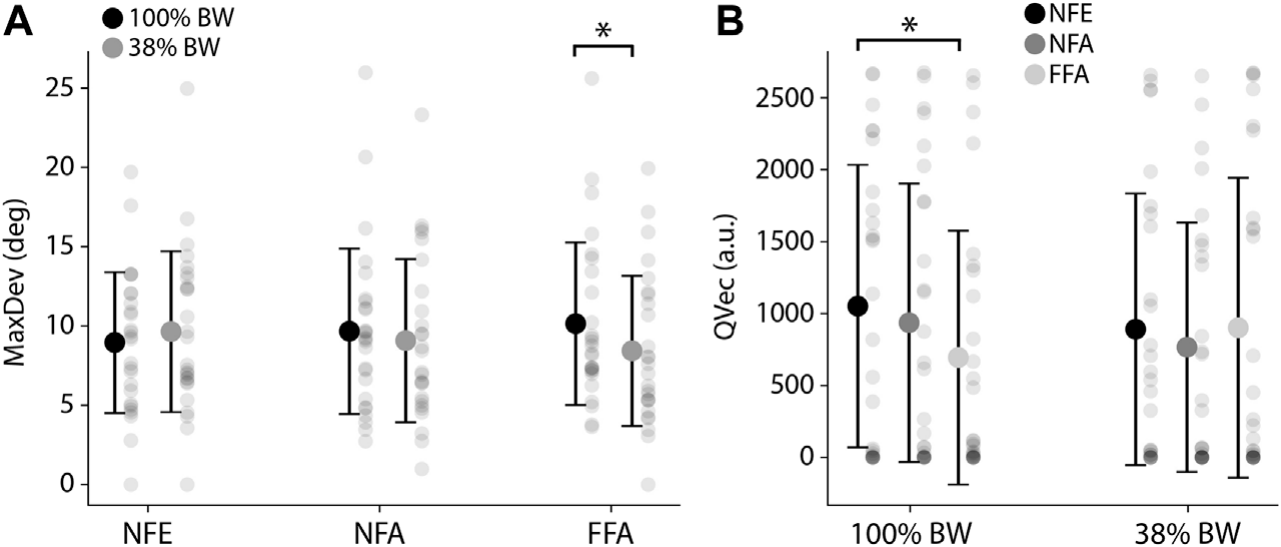
**(A)** MaxDev (Mean maximum deviation of the rod assessed to upright perception) depending on the visual context and the loading condition. *p < 0.05. **(B)** QVec (Mean quantity of vection, i.e., visually-induced forward self-motion perception) depending on the loading condition and the visual context. *p < 0.05.

Regarding upright orientation perception, there was no main effect of the visual condition (F_(2,25)_ = 0.01; *p* = 0.99; ηp^2^ = 0.001) but a significant interaction between PER-loading and the visual conditions (F_(2,25)_ = 5.77; *p* < 0.01; ηp^2^ = 0.19). MaxDev was found to decrease in PER38×FFA as compared to PER100×FFA [−17% (10.1°–8.4°), *p* < 0.05; Figure 6A]. In other words, participants were less influenced by a moving visual background for upright perception following a run at 38% BW compared to 100% BW when the PER-running session presented a coherent visual stimulation (FFA scene).

Considering vection, there was no main effect of the visual condition (F_(2,25)_ = 2.81; *p* = 0.09; ηp^2^ = 0.10) but a significant interaction between PER-loading and the visual condition (F_(2,25)_ = 4.25; *p* < 0.05; ηp^2^ = 0.15). QVec specifically decreased from PER100 × NFE to PER100 × FFA (−34%, *p* < 0.05; Figure 6B). Participants running at 100% BW with a full coherent visual flow (FFA scene) perceived a smaller quantity of self-motion than with no previous visual flow nor head motions allowed (NFE scene). Visuomotor coherence during the PER-runs at 100% BW thus led to a significant decrease of subsequent visually-induced self-motion perception, which is no more apparent at 38% BW.

## 4 Discussion

The main purpose of this study was to examine the interplay between the visual and gravitational contexts and its influence on the adjustments of the running pattern and related spatial perception. Combining LBPPT running with various VR visual scenes showed that the biomechanical parameters of running were not influenced by the visual context, but were affected by the reduction in body weight. Spatial perception, in terms of gravity-related information processing and self-motion perception, exhibited variations depending on the level of simulated gravity but also on the interaction between the latter and the visual context. We will further discuss these observations in the following parts, focusing first on locomotion and spatial perception separately before considering their interaction.

### 4.1 Adaptation of the running pattern

The analysis of the gait at 38% BW revealed the same temporal organization as previously shown at lower unweighting levels (Sainton et al., 2015). Stance time decreased, due to a large increase of flight time, leading to reduce the step frequency as well as active peak force. As expected, such characteristics illustrate a gait that gets closer to skipping, as described by Minetti. (1998), in line with the pattern described at 60% BW by Sainton et al. (2015) and previous studies (Grabowski and Kram., 2008; Smoliga et al., 2015). Grabowski and Kram. (2008) even went down to 25% BW, reporting a decrease in active peak force similar to our findings. Interestingly, they also noted a longer stance time, contrary to our results and other studies conducted in similar settings (Raffalt et al., 2013; Neal et al., 2016).

Yet, in our case, no variation appeared on the stance time components, whatever the visual context. Also, neither braking nor push-off times decreased. However, interindividual variability of the braking phase duration reveals an almost equally Grabowski and Kram. (2008) partitioned behaviour. Some participants modified either the braking time or the push-off time, sometimes both, wheareas the others kept the stance phases constant. This differentiation is new as Sainton et al. (2015) reported a specific decrease in braking time associated to a large inter-individual variability at the larger unweighting level (i.e., 60% BW vs. 80% BW). Unweighting at 38% BW would, thus, introduce a forced choice between two slightly different running patterns depending on what is most efficient for each individual. Adjusting either braking or push-off parts of the stance phase may also reveal a specific control performed online. Here, targeted adaptations may allow for the preservation of the overall efficiency despite the unfamiliar gravitational context. Further analyses of the muscles activity are of course mandatory to deeper investigate the underlying neuromuscular control. In addition, as tests were conducted over a short period (5-min runs), adaptations to the running pattern can be assumed to settle quickly, in line with previous experiments (Sainton et al., 2016). This could be actually the expression of a fast integration of the changes in gravitational context. Alternatively, the visual context could require more time to be taken into account in the adaptive process to unweighting affecting running.

### 4.2 Adaptation of spatial perception

Although visual information did not seem to impact running pattern itself, it did affect spatial perception along with the reduction in body weight. Upright orientation is determined by the importance given to the different available sensory inputs and prior knowledge leading to an estimation of the vertical direction (de Winkel et al., 2018). Following a run at 38% BW, participants were less affected by dynamic visual perturbations during upright estimates than after a run at 100% BW. This points towards a reweighting of sensory inputs required for processing gravity-related information. We suggest here that the importance attributed to sensory inputs involved in graviception is reduced following exposure to simulated Martian gravity, in favor of a higher reliance on the participant’s own body axis as a strong reference for upright perception (de Winkel et al., 2012).

Following this idea, Harris et al. (2017) observed that astronauts exhibit an increase in reliance on body longitudinal axis as a main reference for upright orientation after 10 days in microgravity, and an overall decrease in visual reliance. Additionally, Dyde et al. (2009) showed that background orientation is less important for upright perception in microgravity, which could also provide insights as to why the influence of the moving background in RDT decreases in hypogravity. This study, though, was conducted with static participants and no locomotor activity prior to the test. Our findings suggest that a coherent locomotor activity may help induce a comparable although faster perceptual adaptation under simulated hypogravity.

Notably, such a lower influence of dynamic visual perturbation on the perceived upright orientation specifically occurred after the participants were exposed to visual flow and head motions congruent with the locomotor activity (i.e., FFA condition). This strongly supports the idea that visuomotor coherence during prior exposure to Martian hypogravity is necessary for subsequent changes upon gravity-based processing for upright perception to take place.

Visually-induced self-motion perception, probed through the vection test, was also found to be differently affected by the visual condition, depending on the level of simulated gravity. After a run at 100% BW with full visual flow (FFA condition), vection decreased compared to a run with no visual flow and no possible head-related visual motion (NFE condition). Conversely, this “post-exposure” influence following a run in FFA condition disappeared at 38% BW. Thus, exposure to a coherent visual flow during locomotor activity in normogravity seemed to subsequently reduce the visual influence on self-motion perception, which was not the case after being exposed to a simulated reduced gravity. Some pioneer studies reported an increased visually-induced motion sensation during and after long-term microgravity exposure (Young et al., 1984; Young and Shelhamer, 1990; Young et al., 1992; Watt, 1993; Oman et al., 2000). Noticeably here, while we did not find any main effect of the level of simulated gravity upon subsequent vection (although the trend was barely significant), one may speculate that previous coherent sensorimotor exposure (i.e., FFA condition) may help preserve the sensibility to visually-induced self-motion sensation (which is conversely diminished at 100% BW in our experiment). The time course of these visually-driven sensory reweighting processes is still discussed, since Allison et al. (2012) found a reduction of the ‘oscillation enhancement effect’ on vection sensitivity after short-term microgravity exposure during parabolic flight and a global decrease of visually-induced self-motion perception post flight.

Overall, these fast perceptual adaptations in information processing for upright and self-motion perception following short term locomotor exposure to simulated reduced gravity strongly suggest that prior visual conditions may have a great influence on spatial responses under Mars-like conditions.

### 4.3 Interactions between perceptual and sensorimotor behavior

While visual context was found to interact with body weight variations on spatial perception, it did not influence the temporal and dynamical characteristics of the running pattern which was merely affected by the body weight condition, indicating that sensorimotor and perceptual adaptations are not similarly driven by a same sensory context. Some pioneer studies of Paillard (see Clarac et al., 2009 for a review) reported possible interactions between two levels of action control: the sensorimotor level, which interacts directly with the environment through motor commands and ultimately movement, and the cognitive level, integrating sensory inputs to constantly update internal representations and reference frames. While these two levels communicate and can be influenced by each other, some degree of independence is maintained, allowing one-sided updates and different levels of shared information to external sensory stimulation (Leclere et al., 2022). Our findings supported that claim, in that spatial information processing was rapidly influenced by coherent visual exposure with respect to the locomotor activity being performed, despite the visual condition having no consequence on the observed neuro-mechanical adjustments.

The literature also emphasize how crucial time is for the adaptation of perceptual and sensorimotor levels. Spatial representations are mostly considered rather robust and immune to short term perturbations (Glasauer and Mittelstaedt, 1992; Glasauer and Mittelstaedt, 1998), while motor adaptations have been found very early in novel gravity-related environments (White et al., 2020). For instance, Harris et al. (2017) mentioned that reweighting of visual cues for spatial perception takes days to settle in astronauts on the International Space Station. Here, we provide evidence for distinct adaptive processes governing locomotor behavior and spatial perception in response to short term exposure to a simulated Martian gravity. Strikingly, visuomotor coherence in such an extreme environment might serve to rapidly update spatial cues, and thus optimize perceptual adaptation.

## 5 Conclusion

This study reveals that very short-term locomotor exposure to a simulated Martian gravity (i.e., 5-min runs), using the LBPPT technology, led to specific adaptations of the running pattern and spatial perception. While the former exhibited substantial locomotor changes immune to the visual background, spatial perception was also modified when the visual scene was coherent with respect to the novel sensorimotor context. Of course, one should remain cautious when extrapolating our findings to real locomotion on Mars, due to the existing constraints of the LBPPT analog (e.g., Earth-related vestibular cues, LBPPT ring-related information about the body position with respect to the feet … ). Further experiments including longer periods of exposure may help investigate how the overall sensorimotor context may also differentially affect locomotor activity on Mars, exploring for instance fatigue and the transitions between different unweighting levels on muscular activation and synergies.

## Data Availability

The raw data supporting the conclusion of this article will be made available by the authors, without undue reservation.
